# Accuracy of left ventricular ejection fraction by contemporary multiple gated acquisition scanning in patients with cancer: comparison with cardiovascular magnetic resonance

**DOI:** 10.1186/s12968-017-0348-4

**Published:** 2017-03-24

**Authors:** Hans Huang, Prabhjot S. Nijjar, Jeffrey R. Misialek, Anne Blaes, Nicholas P. Derrico, Felipe Kazmirczak, Igor Klem, Afshin Farzaneh-Far, Chetan Shenoy

**Affiliations:** 1 Department of Medicine, University of Minnesota Medical Center, Minneapolis, MN USA; 2 Cardiovascular Division, Department of Medicine, University of Minnesota Medical Center, 420 Delaware Street SE, MMC 508, Minneapolis, MN 55455 USA; 3 Division of Hematology, Oncology and Transplantation, Department of Medicine, University of Minnesota Medical Center, Minneapolis, MN USA; 4 University of Minnesota Medical School, Minneapolis, MN USA; 5 Duke Cardiovascular Magnetic Resonance Center, Duke University Medical Center, Durham, NC USA; 6 Division of Cardiology, Duke University Medical Center, Durham, NC USA; 7 Division of Cardiology, Department of Medicine, University of Illinois at Chicago, Chicago, IL USA

**Keywords:** Cardiovascular magnetic resonance, MUGA, Cancer, Ejection fraction, Onco-cardiology, Cardio-oncology

## Abstract

**Background:**

Multiple gated acquisition scanning (MUGA) is a common imaging modality for baseline and serial assessment of left ventricular ejection fraction (LVEF) for cardiotoxicity risk assessment prior to, surveillance during, and surveillance after administration of potentially cardiotoxic cancer treatment. The objective of this study was to compare the accuracy of left ventricular ejection fractions (LVEF) obtained by contemporary clinical multiple gated acquisition scans (MUGA) with reference LVEFs from cardiovascular magnetic resonance (CMR) in consecutive patients with cancer.

**Methods:**

In a cross-sectional study, we compared MUGA clinical and CMR reference LVEFs in 75 patients with cancer who had both studies within 30 days. Misclassification was assessed using the two most common thresholds of LVEF used in cardiotoxicity clinical studies and practice: 50 and 55%.

**Results:**

Compared to CMR reference LVEFs, MUGA clinical LVEFs were only lower by a mean of 1.5% (48.5% vs. 50.0%, *p* = 0.17). However, the limits of agreement between MUGA clinical and CMR reference LVEFs were wide at −19.4 to 16.5%. At LVEF thresholds of 50 and 55%, there was misclassification of 35 and 20% of cancer patients, respectively.

**Conclusions:**

MUGA clinical LVEFs are only modestly accurate when compared with CMR reference LVEFs. These data have significant implications on clinical research and patient care of a population with, or at risk for, cardiotoxicity.

## Background

Common cancer treatments such as anthracyclines and trastuzumab are associated with an increased risk of cardiotoxicity, which is responsible for significant mortality and morbidity in cancer survivors [1, 2]. Assessment of left ventricular ejection fraction (LVEF) has been, and continues to be the most widely used method for cardiotoxicity risk assessment prior to, surveillance during, and surveillance after administration of potentially cardiotoxic cancer treatment [3].

Since the 1970s [4], multiple gated acquisition scanning (MUGA) has been one of the first-line imaging modalities for baseline and serial assessment of LVEF for cardiotoxicity. In addition to concerns about exposure to ionizing radiation, there is concern that contemporary gamma cameras may not allow optimal patient positioning for LVEF assessments [3]. Thus, it is possible that contemporary MUGA in cancer patients may not provide accurate estimates of LVEFs. Inaccurate LVEF assessment may carry significant implications for the care of cancer patients receiving potentially cardiotoxic treatment since LVEFs play an important role in decisions to start, continue, hold or stop such treatment.

The objective of this study was to compare the accuracy of LVEFs obtained by contemporary clinical MUGA in consecutive patients with cancer with reference LVEFs from cardiovascular magnetic resonance (CMR), the gold standard technique for assessment of left ventricular volumes and LVEF [5].

## Methods

### Patients and data collection

The study sample consisted of consecutive patients with cancer who had both MUGA and CMR within 30 days between January 2007 and September 2016 at the University of Minnesota Medical Center, Minneapolis, Minnesota, USA. The institutional MUGA database was cross-matched with the University of Minnesota Cardiovascular Magnetic Resonance Registry, an ongoing observational registry including all patients that undergo CMR at the University of Minnesota, to identify the study patients. Patients were excluded if their records indicated any of these intervening clinical events that could potentially impact cardiac function: acute myocardial infarction, heart failure hospitalization, administration of potentially cardiotoxic cancer treatment, or acute systemic illness such as sepsis. An electronic database was created to include demographic information, medical history including reasons for the studies, co-morbidities and medications, and MUGA and CMR findings for each patient. This retrospective cross-sectional study was approved by University of Minnesota’s Institutional Review Board with a waiver of informed consent.

### Multiple-gated acquisition scanning (MUGA)

MUGA scans were performed per standard recommendations to determine LVEF [6, 7] before or after cancer treatment. The UltraTag RBC kit (Mallinckrodt, Inc., St. Louis, Missouri, USA) was used. Erythrocyte labeling was performed using modified in vitro method with technetium 99 m-labeled red blood cells with an activity of approximately 11 to 13 MBq/kg. Images were acquired with a Siemens e-cam dual-head gamma camera (Siemens, Erlangen, Germany) equipped with a parallel hole, low energy high-resolution collimator, with energy window of 15% symmetrically placed over a photopeak of 140 keV. Data were acquired in electrocardiogram-synchronized frame mode using 24 frames per cardiac cycle, with 128 × 128 matrix of 16-bit pixels. Acquisition times were adjusted to achieve a minimum of 200,000 counts per frame. Patients were resting and supine, and the best septal view was individually adjusted from 45° left anterior oblique position with 10°–15° caudal tilt.

Experienced nuclear medicine technologists performed LVEF analyses. Scintigrams were smoothed off-line using standard algorithms, and background correction was performed. The LV regions of interest, as well as background activity, were selected automatically by the computer program (E. Soft; Siemens Medical Solutions, Erlangen, Germany) with manual correction by the interpreting technologists as necessary. Left ventricular time-activity curves were constructed, and LVEF was calculated as ([background-corrected end-diastolic counts − background-corrected end-systolic counts])/(background-corrected end-diastolic counts) × 100. Since our aim was to evaluate “real-world” MUGA data, clinically reported LVEFs were used by design, and analyses were not repeated for this study.

### Cardiovascular magnetic resonance (CMR)

CMR was performed on clinical 1.5 T Siemens scanners (Avanto and Aera) using phased-array receiver coils according to standard recommendations [8, 9]. A typical protocol was as follows: First, localizers were acquired to identify the cardiac position, and the standard long- and short-axes of the heart, and then cine images were acquired in multiple short-axis (every 10 mm to cover the entire LV from the mitral valve plane through the apex) and three long-axis views (2, 3, and 4 chamber) using a steady-state free-precession (SSFP) sequence (repetition time msec/echo time ms, 3.0/1.5; temporal resolution, 35–40 ms; slice thickness, 6 mm; inter-slice gap, 4 mm; flip angle, 60°; in-plane resolution, approximately 1.6 mm × 1.6 mm). Other sequences, including perfusion and delayed-enhancement imaging were performed as clinically indicated.

The LVEF was determined by quantitative analysis according to standard recommendations [10]. To allow use as a reference standard, all CMRs were re-analyzed, blinded to MUGA and clinical data, by a single investigator (C.S.) with 9 years of experience in CMR. Short-axis cine images were used for manual tracing of LV endocardial and epicardial contours at end-diastole and end-systole. Papillary muscles were excluded from the LV myocardial mass (i.e., included in the LV blood volume) to match the methodology used in MUGA. This is an acceptable approach [10], allowing quicker quantitative analyses [11] and is more practical in the routine clinical setting. The LVEF was calculated as ([LV end-diastolic volume − LV end-systolic volume])/(LV end-diastolic volume) × 100.

### Statistical analysis

Statistical analyses were performed on Stata version 13 (StataCorp LP, College Station, Texas, USA). Continuous variables were expressed as means and standard deviations, or medians and inter-quartile ranges (IQR) for data that were not normally distributed. MUGA clinical and CMR reference LVEFs were compared using Student’s paired *t*-test, Bland-Altman analysis and Lin’s concordance correlation coefficient [12]. Lin’s concordance correlation coefficient (*r*_*c*_) provides a measure of reliability based on covariation and correspondence, unlike Pearson’s correlation coefficient (*r*) that provides a measure of linear covariation without accounting for the degree of correspondence between the two sets of values. Pearson’s correlation coefficient (*r*) was used to examine the correlation between the time interval between the two studies and differences between MUGA and CMR LVEFs. We studied the two most common absolute thresholds of LVEF used in cardiotoxicity clinical studies and practice: 50 and 55% [13]. The kappa statistic was used to assess agreement between MUGA and CMR classification of normal vs. abnormal LVEFs. Kappa values were interpreted as widely accepted in the literature [14] – a kappa value of <0 would be considered as less than chance agreement, between 0.01 and 0.20 as slight agreement, between 0.21 and 0.40 as fair agreement, between 0.41 and 0.60 as moderate agreement, between 0.61 and 0.80 as substantial agreement, and between 0.81 and 0.99 as almost perfect agreement. Logistic regression was used to determine univariate predictors of misclassification. All statistical comparisons were two tailed, and a *p* value less than 0.05 was considered to indicate statistical significance.

## Results

### Study sample

Eighty patients were identified as having had MUGA and CMR within 30 days of each other, of which 77 were cancer patients. Two patients received potentially cardiotoxic cancer treatment between MUGA and CMR and were excluded from the study. The remaining 75 patients formed the study cohort. Patient characteristics are listed in Table 1.

**Table 1:** Characteristics of study sample

Age, years	58 ± 12
Male sex	44 (59%)
Body mass index, kg/m^2^	29.5 (26.1, 33.2)
Body surface area, m^2^	2.2 (2.0, 2.4)
Cancer type	
Breast cancer	7 (9%)
Leukemia	23 (31%)
Lymphoma	22 (29%)
Multiple myeloma	13 (17%)
Myelodysplastic syndrome	8 (11%)
Sarcoma	2 (3%)
History of atrial fibrillation	14 (19%)
Left bundle branch block	3 (4%)
Hematocrit at the time of MUGA, %	33.0 ± 5.4
Serum creatinine at the time of MUGA, mg/dL	1.0 ± 0.7
Heart rate during MUGA, beats/min	76.9 ± 14.4
MUGA radioisotope dose, mCi	26.2 ± 1.7
MUGA LVEF, %	48.5 ± 11.5
Interval between MUGA and CMR, days	8.0 (5.5, 10.0)
CMR LV end diastolic volume, mL	167 ± 45
CMR LV end diastolic volume, indexed (mL/m^2^)	76.6 ± 20.0
CMR LV mass, g	121 ± 28
CMR LV mass, indexed (g/m^2^)	55.3 ± 11.6
CMR regional LV dysfunction	6 (8%)
CMR LVEF, %	50.0 ± 9.9

Data are presented as mean ± SD, n (%), or median (interquartile range), unless otherwise indicated

All MUGAs were performed for assessment of LVEF prior to, or after potentially cardiotoxic cancer treatment. CMRs were performed for characterization of cardiomyopathy noted on a prior imaging study (69%), evaluation of suspected obstructive coronary artery disease (15%), evaluation of suspected infiltrative cardiomyopathies (12%), abnormal electrocardiogram (3%), and in one case, evaluation of a suspected intracardiac mass (1%).

### Comparisons between MUGA and CMR LVEFs

There was a median of 8 days (interquartile range 5, 10 days) between MUGA and CMR studies. In 62 (83%) patients, the studies were performed within 15 days of each other. MUGA was performed at least a day before CMR in 69 (92%) patients, the two studies were performed the same day in two (3%) patients, and MUGA was performed after CMR in the remaining four (5%) patients.

**Fig. 1:**
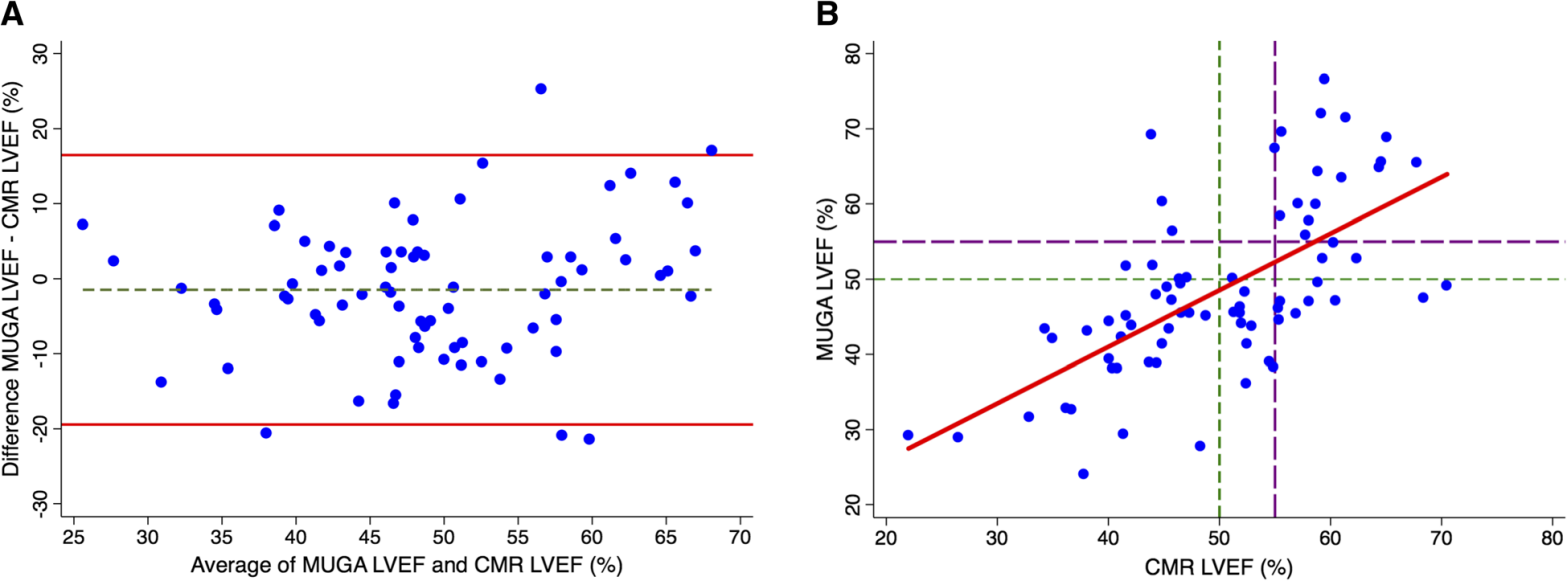
Comparison of MUGA and CMR LVEFs. **a** Bland-Altman plot with limits of agreement and **b** Scatterplot

The mean MUGA LVEF was not significantly different when compared with the mean CMR LVEF (48.5% vs. 50.0%, *p* = 0.17). However, the random error between MUGA and CMR LVEFs was substantial, as evidenced by the Bland-Altman 95% limits of agreement (−19.4, 16.5) (Fig. 1). In 42 (56%) patients, the MUGA LVEF was lower than the CMR LVEF.

The Lin’s concordance correlation coefficient (*r*_*c*_) was 0.63. Using the cutoffs proposed by McBride [15], this indicates poor agreement between MUGA and CMR LVEFs.

There was no correlation between the time interval between MUGA and CMR and the absolute difference between MUGA and CMR LVEFs (*r* = −0.20, *p* = 0.08), or the time interval between the two studies and whether MUGA LVEF was higher or lower than CMR LVEF (*r* = 0.05, *p* = 0.65).

**Fig. 2:**
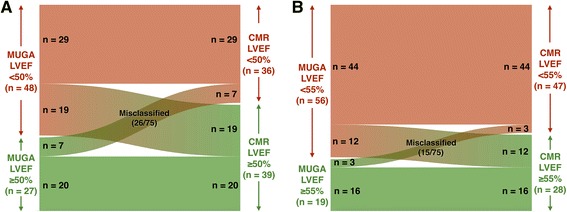
Misclassification of MUGA LVEFs. **a** Threshold of 50% and **b** Threshold of 55%

Using a LVEF threshold of 50%, there was misclassification of 26 of 75 (35%) patients between normal and abnormal categories (Table 2 and Fig. 2) – 19 patients that had MUGA LVEF <50% had a CMR LVEF ≥50%, and seven patients that had MUGA LVEF ≥50% had CMR LVEF <50%. Thus, MUGA LVEFs only had fair agreement with CMR LVEFs (kappa = 0.31).

**Table 2:** Performance of MUGA LVEFs compared with CMR LVEFs at thresholds of 50 and 55%

	LVEF threshold of 50%	LVEF threshold of 55%
Sensitivity	81%	94%
Specificity	51%	57%
False-negative rate	19%	6%
False-positive rate	49%	43%
Positive predictive value	60%	79%
Negative predictive value	74%	84%
Accuracy (correct classification)	65%	80%

Next, using a LVEF threshold of 55%, there was misclassification of 15 of 75 (20%) patients between normal and abnormal categories (Table 2 and Fig. 2) – 12 patients that had MUGA LVEF <55% had a CMR LVEF ≥55%, and three patients that had MUGA LVEF ≥55% had CMR LVEF <55%. MUGA LVEFs had a moderate agreement with CMR reference LVEFs (kappa = 0.54).

**Fig. 3:**
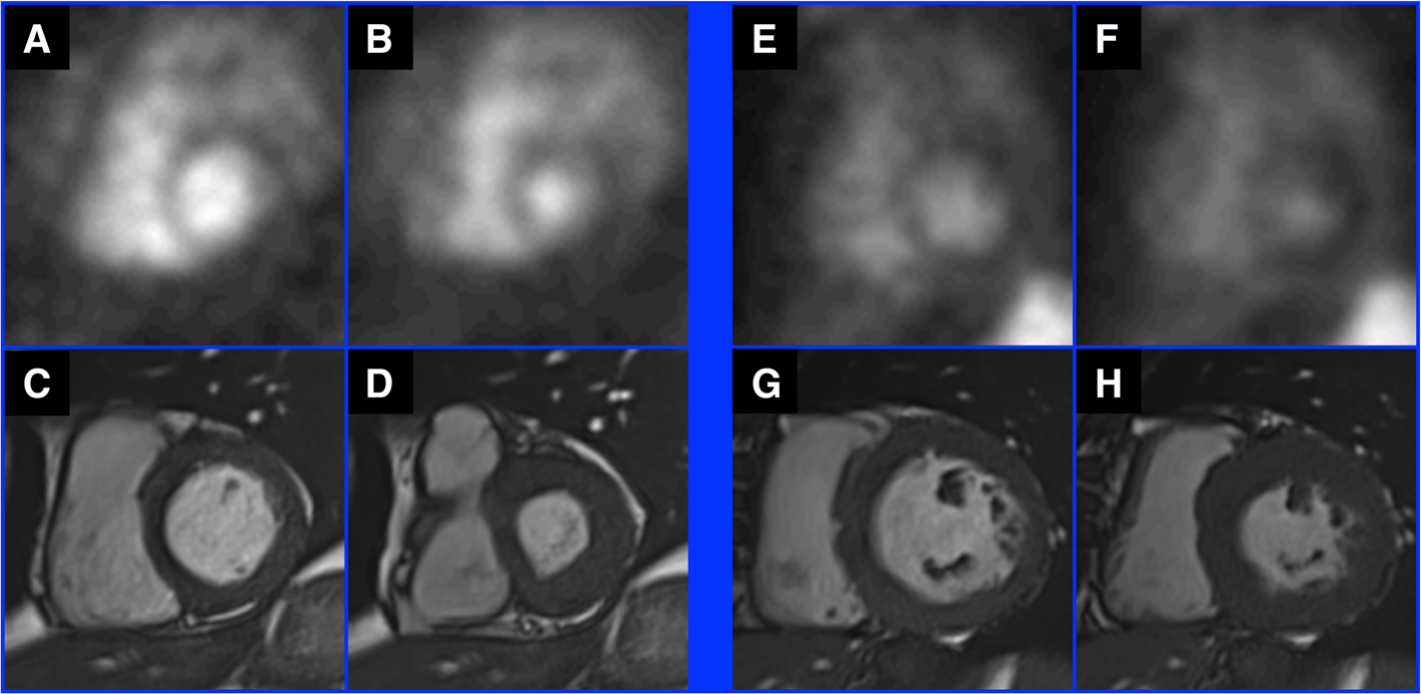
MUGA and CMR images from example study patients with misclassification of MUGA LVEFs. **a** and **b** are end-diastolic and end-systolic MUGA images respectively from a patient whose LVEF was analyzed at 47.0%, **c** and **d** are end-diastolic and end-systolic CMR images respectively from the same patient acquired 4 days later, with LVEF analyzed at 55.2%. **e** and **f** are end-diastolic and end-systolic MUGA images respectively from a patient whose LVEF was analyzed at 69.2%, **g** and **h** are end-diastolic and end-systolic CMR images respectively from the same patient acquired 7 days later, with LVEF analyzed at 44.0%

Figure 3 shows MUGA and CMR images from two study patients with misclassification between MUGA and CMR LVEFs.

### Predictors of misclassification

On univariate logistic regression analysis, the only significant predictor of misclassification at the 50% LVEF threshold was the indexed LV end-diastolic volume (Table 3). At the 55% LVEF threshold, significant predictors of misclassification were indexed LV end-diastolic volume, atrial fibrillation and hematocrit (Table 3). Thus, a smaller indexed LV end-diastolic volume was a predictor of misclassification at both thresholds for a normal LVEF of ≥50% and ≥55%.

**Table 3:** Predictors of misclassification of MUGA LVEFs between normal and abnormal categories compared with CMR LVEFs

Variable	LVEF threshold of 50%		LVEF threshold of 55%	
	Odds ratio (95% CI)	*p* value	Odds ratio (95% CI)	*p* value
Male sex	0.94 (0.36–2.47)	0.90	1.07 (0.34–3.40)	0.91
Body mass index	1.03 (0.95–1.12)	0.50	1.07 (0.96–1.18)	0.22
History of atrial fibrillation	1.54 (0.47–5.03)	0.48	**4.33 (1.21–15.48)**	**0.02**
Left bundle branch block	0.94 (0.08–10.88)	0.96	-	-
Heart rate during MUGA	0.98 (0.95–1.01)	0.26	0.99 (0.95–1.03)	0.54
Hematocrit at the time of MUGA	1.08 (0.99–1.19)	0.10	**1.16 (1.02–1.32)**	**0.02**
Serum creatinine at the time of MUGA	0.34 (0.06–2.06)	0.24	0.48 (0.07–3.26)	0.46
Time interval between MUGA and CMR	0.99 (0.93–1.06)	0.79	0.98 (0.90–1.07)	0.64
CMR LV end diastolic volume, indexed	**0.97 (0.94–0.99)**	**0.03**	**0.96 (0.92–0.99)**	**0.04**
CMR LV mass, indexed	0.98 (0.94–1.03)	0.46	0.98 (0.93–1.03)	0.43
CMR regional LV dysfunction	0.35 (0.04–3.18)	0.35	-	-

## Discussion

Using a sample of cancer patients that had both MUGA and CMR performed within 30 days, we found that MUGA LVEFs were only modestly accurate when compared with reference LVEFs by CMR, the gold standard technique for the assessment of LVEF. MUGA LVEFs were systemically lower by 1.5%, suggesting only a small *mean* discordance between the two methods. However, the limits of agreement between MUGA and CMR LVEFs were wide (−19 to 16%). This suggests large *individual* discordance, or in other words, low accuracy for MUGA LVEFs when compared to CMR LVEFs. Furthermore, using LVEF thresholds of ≥50% and ≥55% to define normal, there was misclassification between MUGA and CMR LVEFs in categorizing 35 and 20% of patients respectively.

Schwartz et al. in 1987 demonstrated in patients receiving doxorubicin that LVEF estimates by MUGA used per their proposed guidelines reduced the incidence and severity of clinical congestive heart failure [16]. Normal LVEF was defined as ≥50%, and cardiotoxicity was defined as an absolute decrease in LVEF of ≥10% with a final LVEF of ≤50% [16]. These data, along with data demonstrating high reproducibility [17] and low variability [18], established MUGA as the modality of choice for serial testing of LVEF in patients with cancer. However, validation of the accuracy of MUGA in these studies was done with comparisons to contrast left ventriculography [19], which has significant variability [20], and is arguably a poor reference standard. In a phantom study comparing CMR, MUGA, and left ventriculography, left ventriculography was the least accurate, and MUGA was less accurate than CMR [21]. Of note, CMR in this study was performed using a gradient-echo sequence, which has lower blood-to-myocardium contrast [22], accuracy and reproducibility than the currently used SSFP sequence [23]. Furthermore, in an in vitro model, LV volumes by CMR have been shown to be highly accurate when compared to volumes obtained using latex casts of excised human LVs [24].

The systematic bias between CMR and MUGA LVEFs has been variable in the literature [5, 25, 26]. The discrepancies are likely due to differences between institutions in imaging and analysis techniques, and software. Audits of MUGA LVEFs in the United Kingdom, Australia and New Zealand have demonstrated significant variability between centers, mainly due to differences in the software used for LVEF analyses [27, 28]. The systemic bias between mean MUGA and CMR LVEFs in this study is overshadowed by the substantial random error when comparing the two modalities. Possible sources of inaccuracy associated with MUGA include suboptimal patient positioning with current gamma cameras, limited spatial resolution, the need for background correction, errors from overlapping structures, and gating inaccuracies due to arrhythmias.

Our findings are in contrast with those of Walker et al. [29] who studied 50 consecutive patients with breast cancer prior to adjuvant trastuzumab, at 6 and 12 months and found a strong correlation (*r* of 0.88, 0.97 and 0.87 at baseline, 6 and 12 months respectively) between MUGA and CMR. It could be argued that this study was not reflective of real-life practice as demonstrated by the exclusion of patients with a history of atrial fibrillation or intraventricular conduction delay. Although consecutive patients were enrolled in the study, a majority had LVEF <55% at baseline (prior to trastuzumab therapy) and proceeded to receive trastuzumab. Additionally, whether MUGA and CMR LVEF analyses were blinded to the results of the other imaging technique was not stated.

Early MUGA studies in the 1970s and 1980s were performed using small-field-of-view, single-headed gamma cameras that allowed optimal positioning of the patient to obtain the best separation between the left and the right ventricles. Current gamma cameras are predominantly large-field-of-view, dual-headed systems that do not permit this degree of patient positioning [3].

We attempted to identify potential associations for misclassification between MUGA and CMR LVEFs in our cohort. Smaller LV size was a significant predictor of misclassification, which we speculate is a consequence of differences in spatial resolution between the two modalities. Smaller hearts may have less accurate LVEF measurements by MUGA due to its relatively lower spatial resolution. The presence of arrhythmias may have also contributed to discrepancies between the two imaging modalities, as a history of atrial fibrillation was a significant predictor of misclassification at the higher LVEF threshold.

Our findings have important implications for clinical investigations and the care of patients receiving potentially cardiotoxic cancer treatment. MUGA is frequently used in these patients; in a study of 2203 patients 66 years or older who received trastuzumab for adjuvant treatment of breast cancer, 28% had baseline and serial assessment of LVEF with MUGA alone, and 23% with a combination of MUGA and echocardiography [30]. Important therapeutic decisions are often based on the LVEF in patients with cancer. Imprecise LVEFs leading to incorrect classification of patients as normal or abnormal may lead to erroneous decisions about the choice of standard-of-care treatment, or less cardiotoxic – but potentially less effective – alternatives such as reduced doses of standard chemotherapy regimens or nonstandard regimens. Similarly, they may influence incorrect decisions regarding the frequency of clinical follow up, screening by imaging to detect cardiotoxicity, and treatment with cardiac medications for the cardiomyopathy. Ultimately, erroneous classification of patients as normal or abnormal due to inaccurate LVEFs may result either in cardiomyopathy and heart failure that could potentially have been prevented, or lower treatment response and worse cancer outcomes from less effective cancer treatment used in response to unwarranted concerns for cardiotoxicity.

MUGA involves the use of ionizing radiation in a patient population that requires serial studies. Guidelines for cardiac monitoring after trastuzumab treatment recommend the use of the same imaging modality throughout the course of treatment [31, 32]. A breast cancer patient receiving adjuvant trastuzumab is recommended to have LVEF assessment before starting treatment, every 3 months during, upon completion of treatment, and every 6 months for at least 2 years following completion of treatment [33]. More frequent monitoring is recommended if trastuzumab is withheld for a significant drop in LVEF [33]. With 12 months of adjuvant trastuzumab as the standard of care, this translates into a minimum of nine studies. With an average typical effective ionizing radiation dose of 8 mSv per MUGA [34, 35], the use of MUGA would result in a significant dose of ionizing radiation with associated risks of radiation-related secondary cancers [36, 37]. A recent publication highlighted this issue through the case of a patient with multiple myeloma who received 17 MUGAs, corresponding to an effective radiation dose of 113 mSv, over a span of 3 years [38]. A scientific statement from the American Heart Association on approaches to enhancing radiation safety in cardiovascular imaging carries the recommendation that when a cardiac imaging study is indicated, a comparable test with similar accuracy, cost and convenience, which does not use ionizing radiation, should be preferred [39].

Echocardiography and CMR are alternatives that do not involve ionizing radiation. However, two-dimensional echocardiography has been shown to have limited performance compared to CMR for the detection of cardiotoxicity in adult survivors of childhood cancer for cardiomyopathy [40]. Unlike MUGA, CMR provides additional clinically valuable information including assessments of right ventricular (RV) size and function, atrial size and function, valvular disease, pericardial disease, intracardiac thrombus and extracardiac pathology. In our study, CMR revealed that 80% of patients had at least one additional abnormality: 52% had RV dysfunction (defined as RVEF <50%), 33% had an enlarged left atrium, 12% had an enlarged right atrium, 13% had significant valvular disease, 9% had a pericardial effusion, 11% had pleural effusions, 5% had an intracardiac thrombus, and 3% had cardiac tumors. Additionally, late gadolinium enhancement CMR has the ability to detect the presence and patterns of fibrosis, which would help identify the etiology for cardiomyopathy [41] in cancer patients. T1 mapping is a newer CMR technique for the detection of diffuse myocardial disease, that holds significant promise in the prediction, early detection and prognostication of cardiotoxicity [42]. Thus, findings on CMR other than the LVEF may have significant impact on management and clinical decision-making [43].

### Limitations

Our findings must be interpreted in the context of the study design. Rather than to perform a head-to-head comparison of two imaging modalities, our aim was to examine the accuracy of real world, clinical LVEFs by MUGA, which oncologists and cardiologists use every day in clinical practice to make important decisions. To achieve this, we compared clinically analyzed MUGA LVEFs with CMR LVEFs that were ascertained by a single blinded expert investigator for this study. This design allowed comparison of real world MUGA LVEFs to arguably the most accurate estimates possible, of the true LVEF (since even a necropsy cannot provide a LVEF).

Since MUGA and CMR studies were not performed on the same day in all cases except one, there is a possibility of true LVEF changes in the interim period. We limited this possibility by excluding patients with clinical events and potentially cardiotoxic treatment during or in the time interval between the two studies. Additionally, we did not find correlations between the time interval between the two techniques and the absolute difference in LVEFs, whether MUGA clinical LVEF was higher or lower than CMR reference LVEF, or whether there was misclassification between normal and abnormal categories.

While making clinical decisions on the management of cardiotoxicity, the change in LVEF is often used in conjunction with the absolute LVEF. We did not investigate changes in LVEF in this study. Finally, this is a relatively small, single-center study subject to referral bias.

## Conclusions

MUGA LVEFs are only modestly accurate when compared with reference LVEFs from CMR. At LVEF thresholds of 50 and 55%, there is misclassification of 35 and 20% of cancer patients, respectively, to either normal or abnormal categories. Given the significant implications of these hypothesis-generating data on clinical research and patient care of a population with, or at risk for, cardiotoxicity, prospective comparisons of MUGA with CMR for the management of cancer patients are urgently warranted.

## Abbreviations

CMR: Cardiovascular magnetic resonance
LV: Left ventricle
LVEF: Left ventricular ejection fraction
MUGA: Multiple gated acquisition scanning
SSFP: Steady State Free Precession
